# Hospital distribution, seasonality, time trends and antifungal susceptibility profiles of all *Aspergillus* species isolated from clinical samples from 2015 to 2022 in a tertiary care hospital

**DOI:** 10.1186/s12866-024-03267-8

**Published:** 2024-04-03

**Authors:** Iacopo Franconi, Cosmeri Rizzato, Emilia Ghelardi, Antonella Lupetti

**Affiliations:** 1 Department of Translational Research and New Technologies in Medicine and Surgery, University of Pisa, Via San Zeno 37-39, 56127 Pisa, Italy; 2 Mycology Unit, Pisa University Hospital, Pisa, Italy; 3 Department of Biology, University of Pisa, Pisa, Italy

**Keywords:** *Aspergillus* spp., Prevalence, Incidence, Species distribution, Antifungal compounds, MIC, Epidemiological Cut-off Values, CLSI

## Abstract

**Background:**

*Aspergillus* species cause a variety of serious clinical conditions with increasing trend in antifungal resistance. The present study aimed at evaluating hospital epidemiology and antifungal susceptibility of all isolates recorded in our clinical database since its implementation.

**Methods:**

Data on date of isolation, biological samples, patients’ age and sex, clinical settings, and antifungal susceptibility tests for all *Aspergillus* spp. isolated from 2015 to 2022 were extracted from the clinical database. Score test for trend of odds, non-parametric Mann Kendall trend test and logistic regression analysis were used to analyze prevalence, incidence, and seasonality of *Aspergillus* spp. isolates.

**Results:**

A total of 1126 *Aspergillus* spp. isolates were evaluated. *A. fumigatus* was the most prevalent (44.1%) followed by *A. niger* (22.3%), *A. flavus* (17.7%) and *A. terreus* (10.6%). *A. niger* prevalence increased over time in intensive care units (*p*-trend = 0.0051). Overall, 16 (1.5%) were not susceptible to one azole compound, and 108 (10.9%) to amphotericin B, with *A. niger* showing the highest percentage (21.9%). The risk of detecting *A. fumigatus* was higher in June, (OR = 2.14, 95% CI [1.16; 3.98] *p* = 0.016) and reduced during September (OR = 0.48, 95% CI [0.27; 0.87] *p* = 0.015) and October as compared to January (OR = 0.39, 95% CI [0.21; 0.70] *p* = 0.002. *A. niger* showed a reduced risk of isolation from all clinical samples in the month of June as compared to January (OR = 0.34, 95% CI [0.14; 0.79] *p* = 0.012). Seasonal trend for *A. flavus* showed a higher risk of detection in September (OR = 2.7, 95% CI [1.18; 6.18] *p* = 0.019), October (OR = 2.32, 95% CI [1.01; 5.35] *p* = 0.048) and November (OR = 2.42, 95% CI [1.01; 5.79] *p* = 0.047) as compared to January.

**Conclusions:**

This is the first study to analyze, at once, data regarding prevalence, time trends, seasonality, species distribution and antifungal susceptibility profiles of all *Aspergillus* spp. isolates over a 8-year period in a tertiary care center. Surprisingly no increase in azole resistance was observed over time.

**Supplementary Information:**

The online version contains supplementary material available at 10.1186/s12866-024-03267-8.

## Background

*Aspergillus* species are a group of environmental molds that belong to the phylum of Ascomycota [1, 2]. Several hundreds of *Aspergillus* species have been discovered to date [3], and the number of species reported to be associated with human infections in both immunocompetent and immunocompromised are on the rise [2, 4, 5]. Within the *Aspergillus* genus 4 subgenera have been described so far [6, 7]. In recent years with the advances in the molecular diagnostic field another group of *Aspergillus* species has been identified, falling under the name of “Cryptic Aspergillosis” [4, 6, 7]. With the implementation of Matrix Assisted Laser Desorption Ionization Time-Of-Flight (MALDI-TOF) mass spectrometry newer species belonging to this group have been defined, such as *Aspergillus lentulus, Aspergillus thermomutans, A. sydowii* [6–8]*.* Among all *Aspergillus* species, *Aspergillus fumigatus* remains the most frequently isolated in clinical practice [9, 10], followed by *Aspergillus flavus*, *Aspergillus niger* and *Aspergillus terreus* [11, 12].

Clinical manifestations of *Aspergillus* infections vary upon host’s preexisting conditions [2, 13, 14]. Cystic fibrosis patients, for example, are at high risk of developing allergic bronchopulmonary aspergillosis [2]. In immunocompetent hosts *Aspergillus* spp. infections usually involve preformed lung cavities evolving in a chronic non-invasive form. This condition is found in sarcoidosis, lung abscess, pneumocystosis, and subjects with tuberculosis caverns [2, 15]. It is commonly reported as fungus ball, or “aspergilloma” [16]. This clinical condition is usually asymptomatic and tends to occur, as previously mentioned, mainly in a pre-formed lung cavity where inflammatory and granulomatous reactions enucleate the fungal infectious process [2, 17]. Other forms of non-invasive aspergillosis in the immunocompetent host are now classified as chronic pulmonary aspergillosis [10, 15, 17, 18], and are correlated with a variety of different medical pictures from sub-clinical and/or mild clinical manifestations as constitutional symptoms, cough, loss of weight, to hemoptysis and even dyspnea [15, 18]. Global surveys estimate that more than 350.000 cases of chronic pulmonary aspergillosis are diagnosed annually in recently tuberculosis-treated patients (within 12 months since end of treatment) [2, 15].

Despite being a serious and even difficult to treat medical condition, chronic pulmonary aspergillosis is neither the most common clinical manifestation nor the most aggressive form of *Aspergillus* infections [13, 19]. Invasive Aspergillosis (IA) is the most important clinical issue related to *Aspergillus* spp. infections [2, 10, 19]. Clinical manifestations differ greatly from chronic pulmonary aspergillosis, as IA is associated with angioinvasion and rapid progressive disease with poor prognosis if treatment is not promptly started [10, 19]. First localization and associated symptoms usually start from the respiratory system and show high tendency of dissemination to other sites such as skin, eye and central nervous system [19]. It has been estimated that *Aspergillus* invasive infections affect more than 300.000 subjects worldwide per year and are associated with high mortality rates ranging from 45 to 99% [13]. This fact is closely related to the increase in the number of subjects at risk of acquiring and developing IA. Immunocompromised hosts are the target of invasive fungal infections, in particular IA. Among them, neutropenic patients, subjects undergoing solid organ and/or hematopoietic stem-cell transplantation presented the highest risk of acquiring IA [13, 20]. Secondary, all patients undergoing chronic immunosuppressive treatments with corticosteroids, or diagnosed with malignancies, or with chronic diseases as end-stage liver or kidney disease, severe COPD (Chronic Obstructive Pulmonary Disease) are at risk of developing IA [13]. Another factor associated with IA is an underlying critical condition requiring recovery in the intensive care units (ICUs) and invasive ventilation [21], and recently even severe COVID-19 [22].

Despite recent developments in the molecular diagnostic field, phenotypic characteristics and both macroscopic and microscopic examination remain the fundamentals of routine identification and detection of *Aspergillus* spp. in most clinical microbiology laboratories [23]. MALDI-TOF analysis, 18S DNA sequencing and multiplex PCR have proved to be resourceful tools in establishing the diagnosis at a species level and their use in clinical practice is therefore increasing [5, 24, 25]. However, despite their sensitivity, specificity and accuracy, such methods might be expensive, time consuming and require adequate laboratory expertise and personnel. In addition, the spectrum of detection and referral libraries might not include all cryptic species and protein extraction procedures still have to be standardized [23]. To this point it is important to mention that a proven diagnosis of IA can only be obtained through microscopic histologic examination with the recovery of fungal structures within the collected tissue specimen along with signs of tissue damage in the surrounding area [10, 26]. Microbiological examinations, host’s predisposing clinical conditions, serology and suggesting imaging can only drive probable diagnosis of IA [10, 26].

The WHO has recently listed *Aspergillus fumigatus* among the fungal priority pathogens list due to its mortality rates, the prolonged hospital stay associated with IA, and its increasing antifungal resistance rates, mainly for azole compounds due to point mutations in the *Cyp51A* gene [27–31]. Experts always recommend to screen for azole resistance and to perform therefore antifungal susceptibility testing [27]. Such tests rely on an in-vitro broth microdilution method performed into 96-well-plates and have been standardized by both the Clinical and Laboratory Standards Institute (CLSI) [32] and the European Committee on Antimicrobial Susceptibility Testing (EUCAST) [33]. Even if CLSI referral document [34] for the interpretation of antifungal Minimum Inhibitory Concentration (MIC) does not provide Clinical Breakpoints (CBP) for the tested drugs, interpretation of the broth microdilution test has been carried on with the use of Epidemiological Cut-off Values (ECV) [34–38]. ECVs are calculated through the analysis of MIC distributions for both wild-type (WT) and non-wild-type *Aspergillus* species. WT strains do not harbor any acquired resistance for the tested drug. ECV is set from the MIC value of two-fold dilution above the mode of MIC of WT strains [38]. Therefore, non-WT isolates are identified as a fungal strain with a MIC above the ECV [38] and could be related to isolates with acquired resistance.

In consideration of the worrisome clinical burden of IA, the reported increase in azole resistance and the need of clinical surveillance studies reported by the WHO document, the present study aimed at evaluating prevalence, time trends, seasonality, species distribution and antifungal susceptibility profiles of all *Aspergillus* spp. isolates recovered in our clinical database from its implementation in 2015 to 2022.

## Methods

This is a retrospective surveillance study conducted at Pisa University Hospital, Mycology Unit. The teaching hospital is a tertiary level care facility that hosted 1108 beds and witnessed 49045 admissions in 2021 with total patient-days of 280939 for the same year. Authors conducted the research searching through the clinical microbiology database from January 1st, 2015 to December 31st, 2022. All recorded *Aspergillus* spp. isolates for which an antifungal susceptibility test was performed were included in the analysis. In order to avoid potential biases when considering multiple isolates from the same subjects, it was decided to consider only the first positive sample culture and associated antifungal susceptibility test over a time span of 30 days for each *Aspergillus* species detected as reported for other microorganisms [39, 40], in order to avoid repetitions and alteration of results. Data regarding patients’ ID were not included in the dataset, so patients were anonymized, and each subject was identified with a specific code that had no relation with private patients’ information. Extracted variables included: age, sex, clinical setting, biological sample, date of detection, and species of *Aspergillus*, date of identification, and antifungal compounds susceptibility test with punctual MICs. Due to the large span of clinical settings, that ranged between 30 to 40 clinical wards in the hospital during the 8-year study period, authors decided to recode clinical wards according to the branch of medical specialties as reported in other studies for other fungal pathogens in the following list: internal medicine, surgery, intensive care unit, and outpatient clinic [39, 40]. Outpatient clinic was composed by the following day hospital and outpatient services: pneumology, infectious diseases, pediatrics and family medicine answering all clinical needs of patients that were not currently admitted to the hospital. Incidence of *Aspergillus* isolation per 10.000 patient-day was calculated for each *Aspergillus* species, total patient-day data per year were obtained from hospital publicly available repository (https://www.ao-pisa.toscana.it/index.php?option=com_content&view=article&id=5650:relazione-sanitaria-2021&catid=264&Itemid=112).

### *Aspergillus* spp. isolation and identification

Molds isolates were recovered from all clinical samples previously cultured on chloramphenicol and gentamycin added Sabouraud agar medium in aerobic atmosphere at 37 °C or directly at 30 °C for 48 h. Each suspected mold was then sub-cultured on chloramphenicol and gentamicin added Sabouraud agar, in order to obtain a purified culture of the *Aspergillus* spp. and incubated at 30 °C for 72 h in aerobic atmosphere. First, fungal identification was made through macroscopic and microscopic examination of the phenotypic characteristics of the recovered mold [5, 23]. In addition, molecular approach to diagnosis has been performed by MALDI-TOF since 2020 [41]. Species other than *A. fumigatus*, *A. niger*, *A. flavus*, and *A. terreus* were classified as Less-Frequently Isolated *Aspergillus* species, (LFI-*Aspergillus* spp.)

### Antifungal susceptibility test

*Aspergillus* spp. inoculum suspensions were prepared in sterile 0.9% NaCl with 0.1% Tween 20 as reported in the CLSI M38-A2 document [32]. The solution was homogenized by vortex to obtain an inoculum of 0.4–3.3 × 10^6^ colony-forming units (CFU)/mL of spore suspension. This spore suspension was then resuspended in RPMI 1640 with 0.2% glucose to reach a final concentration of 0.4—0.5 × 10^4^ CFU/mL in each well of the 96-well microdilution plate [32]. Colorimetric broth microtitration test was performed using Sensititre YeastOne Y010© 96-well microdilution plates (ThermoFisher Scientific, Waltham, Massachusetts, U.S.). Next, these plates were incubated at 30 °C and checked for fungal growth at 24 h, 48 h, and 72 h according to different species. All tested molecules at the broth microdilution method that showed a MIC above the ECV pattern were then retested with E-test. Tested antifungal compounds analyzed in this study were voriconazole, posaconazole, itraconazole and amphotericin B. Since Sensititre YeastOne Y010© referred to the CLSI M57S reference standard, MIC values were analyzed and interpreted accordingly. Microbiological resistance is associated with clinical implications as therapeutic correlations, like treatment failure, and it is determined by comparing MIC values with clinical breakpoints. CLSI guidance documents did not provide Clinical Breakpoints (CBP) for antifungal compounds, but reported only ECV, which allowed the clinical microbiologist to regroup *Aspergillus* spp. isolates between “wild-type” (WT) and “non-wild-type” (non-WT) [35–38]. ECVs values include 95% of all MIC values reported for the WT mold population. Consequently, even if mold isolates resistant to antifungal compounds show MIC values above the ECVs, the “wild-type” and “non-wild-type” terms and definitions could only reflect epidemiological investigations and distinctions as ECVs cannot be used exactly as CBPs [35, 37, 38]. Therefore, in this study, references to antifungal susceptibility profiles of *Aspergillus* spp. will only address presence of a WT or non-WT phenotype. *Aspergillus fumigatus* isolates that had MIC values ≤ 1 mg/L for itraconazole, ≤ 1 mg/L for voriconazole, ≤ 0.25 mg/L for posaconazole, ≤ 2 mg/L for amphotericin B were considered WT for the reported antifungal compounds [34]. *Aspergillus terreus* isolates that had MIC values ≤ 2 mg/L for itraconazole, ≤ 2 mg/L for voriconazole, ≤ 1 mg/L for posaconazole were considered WT for the reported antifungal compounds. According to the CLSI M57S document, *A. terreus* ECV for amphotericin B is 4 mg/L however, the document states that a reduced MIC for such antifungal compound in this species does not correlate with positive clinical outcomes as wild type MICs for this molecule have not been yet defined. *Aspergillus niger* isolates that had MIC values ≤ 4 mg/L for itraconazole, ≤ 2 mg/L for voriconazole, ≤ 2 mg/L for posaconazole and ≤ 2 mg/L for amphotericin B were considered WT for the reported antifungal compounds. *Aspergillus flavus* isolates that had MIC values ≤ 1 mg/L for itraconazole, ≤ 2 mg/L for voriconazole, ≤ 0.5 mg/L for posaconazole and ≤ 4 mg/L for amphotericin B were considered WT for the reported antifungal compounds [34].

## Statistical analyses

Non-parametric Mann Kendall trend test was used to assess variations in incidence rates overall and according to each species as reported in the study of Goemaere and colleagues [41]. Additionally, confirmatory linear regression model was fit to evaluate trends in incidence of isolates over time. Score test for trends of odds was used to assess whether the prevalence of each *Aspergillus* species increased over-time overall and in relation to clinical settings. Logistic regression analyses adjusted for age, sex, and year of study were performed to evaluate risk of detection of each *Aspergillus* species according to clinical settings. Authors defined internal medicine as the reference standard to establish multiple comparisons at logistic regression. Same logistic regression analyses were performed to investigate trends in seasonal frequency of each *Aspergillus* species, authors decided to set the first month of the year as the reference standard for comparison. All statistical analyses were performed using STATA/IC 16.1 for Windows© (StataCorp LLC 4905 Lakeway Drive College Station, Texas 77,845–4512, USA).

## Results

A total of 1126 *Aspergillus* spp. isolates were evaluated, belonging to 14 different *Aspergillus* species. Mean age of study population was 56 ± 21, median age was 60, interquartile range (IQR = 40–74 years). Male subjects were 707 (62.79%), female 418 (37.12%). Over the 8-year study period, the number of *Aspergillus* species isolated according to clinical settings and samples and overall prevalence is reported in Table 1: 383 (34%) *Aspergillus* spp. isolates were recovered in the outpatient clinic, 333 (29.7%) in internal medicine, 322 (28.6%) in intensive care unit, and 88 (7.8%) in surgery. *Aspergillus* spp. were isolated from the following clinical samples: sputum 521 (46.3%), bronchoalveolar lavage fluid (BAL) 448 (39.8%), wound/biopsy 57 (5.1%), nasal swab 42 (3.7%), ear swab 39 (3.5%), pleural fluid 14 (1.2%), sinus discharge 3 (0.3%), cerebro-spinal fluid (CSF) 1 (0.08%), eye swab 1 (0.08%).

**Table 1:** Overall number of *Aspergillus* species isolated according to clinical settings and samples

Clinical setting	N° of Isolates (%)					
	***A. fumigatus***	***A. niger***	***A. flavus***	***A. terreus***	**Others**^**a**^	**Overall**
**Internal Medicine**	169 (50.7%)	58 (17.4%)	58 (17.4%)	36 (10.8%)	12 (3.6%)	333
**Surgery**	35 (39.8%)	25 (28.4%)	16 (18.2%)	4 (4%)	8 (9%)	88
**Intensive care**	123 (38.2%)	66 (20.5%)	66 (20.5%)	54 (16.7%)	13 (4%)	322
**Outpatient clinic**	170 (44.4%)	102 (26.6%)	59 (15.4%)	25 (6.5%)	27 (7%)	383
**Sputum**	254 (48.7%)	120 (23%)	82 (15.7%)	35 (6.7%)	30 (5.7%)	521
**BAL**	186 (41.5%)	87 (19.4%)	80 (17.9%)	76 (16.9%)	19 (4.2%)	448
**Wound/biopsy**	29 (50.9%)	14 (24.6%)	7 (12.3%)	5 (8.8%)	2 (3.5%)	57
**Ear swab**	6 (15.4%)	16 (41%)	14 (35.9%)	1 (2.6%)	2 (5.1%)	39
**Nasal swab**	10 (23.9%)	13 (30.9%)	12 (28.6%)	2 (4.7%)	5 (11.9%)	42
**Sinus discharge**	1 (33.3%)	1 (33.3%)	1 (33.3%)	0	0	3
**Pleural fluid**	10 (71.4%)	0	2 (14.3%)	0	2 (14,3%)	14
**CSF**	0	0	1 (100%)	0	0	1
**Eye swab**	1 (100%)	0	0	0	0	1
**total**	497 (44.1%)	251 (22.3%)	199 (17.7%)	119 (10.6%)	60 (5,3%)	1126

^a^Others include: *Aspergillus nidulans*, *Aspergillus candidus*, *A*. *sclerotiorum*, *A*. *versicolor*, *A. lentulus*, *A. glaucus*, *A*. *usutus*, *A. oryzae*, *A*. *clavatus*, *A*. *ochraceus*

No missing value was reported for voriconazole, while missing values for the other antifungal compounds were as follows: 10 for amphotericin B (0.8%), 18 (1.6%) for posaconazole, 170 (15.1%) for itraconazole. Overall, 16 (1.5%) clinical isolates had a MIC value above the ECVs for at least one azole compound and 108 (10.8%) for amphotericin B. No isolate had MIC values above ECVs for all the tested azoles together. All non-WT phenotypes exceeding the ECVs were confirmed at E-test.

### Hospital *Aspergillus* species distribution

All the recovered *Aspergillus* spp. are reported as follows according to overall prevalence: *Aspergillus fumigatus* 497 (44.1%), *Aspergillus niger* 251 (22.3%), *Aspergillus flavus* 199 (17.7%), *Aspergillus terreus* 119 (10.6%), others 60 (5.3%). Others comprises the following species: *A. nidulans* 23, *A. candidus* 10, *A. sclerotiorum* 8, *A. versicolor* 7, *A. lentulus* 6, *A. glaucus* 2, *A. usutus* 1, *A. oryzae* 1, *A. clavatus* 1, and *A. ochraceus* 1. *A. fumigatus* was the most prevalent isolate overall and according to clinical settings, 169 (50.7%) out of the 333 were isolated in internal medicine, 35 (39.8%) out of 88 in surgery, 123 (38.2%) out of 322 in ICU, and 170 (44.4%) out of 383 in the outpatient clinic, Table 1. At logistic regression analysis risk of detecting *A. niger* was lower in surgery (OR = 0.34, 95% CI [0.14; 0.79] *p* = 0.012) and higher in the outpatient clinic (OR = 1.72, 95% CI [1.19; 2.47] *p* = 0.005), and risk of detecting *A. terreus* was higher in intensive care unit (OR = 1.66, 95% CI [1.06; 2.61] *p* = 0.028) and lower in the outpatient clinic (OR = 0.58, 95% CI [0.34; 0.17] *p* = 0.043) as compared to internal medicine. For LFI-*Aspergillus* spp. risk of detection was higher in surgery (OR = 2.72, 95% CI [1.07; 6.93] *p* = 0.036). No different risk of detecting *A. flavus* according to clinical settings was found.

According to biological samples *A. fumigatus* was still the most prevalent isolate in sputum (48.7% of all positive sputum samples), BAL (41.5%), wound/biopsy (50.9%), pleural fluid (71.4%) and eye swab (100%). On the contrary, *A. niger* resulted to be the most prevalent in ear swab (41%) and nasal swab (30.9%), followed by *A. flavus* in ear swab (35.9%) and nasal swab (28.6%). *A. terreus* was mainly isolated from BAL.

### Seasonality

**Fig. 1:**
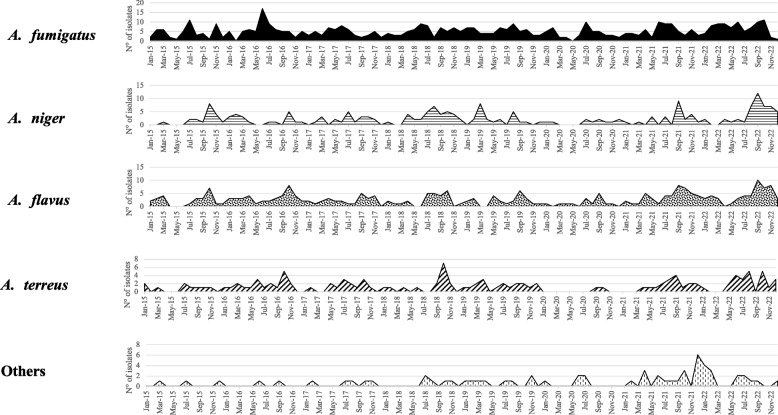
Monthly prevalence of the 4 most commonly reported and Less-Frequently-Isolated *Aspergillus* species. *A. fumigatus* is reported in full black color, *A. niger* horizontal bars, *A. flavus* black dots, and *A terreus* in diagonal bars, Other *Aspergillus* species are reported with vertical segmented lines

Monthly seasonality of *Aspergillus* species isolation across the 8-year study period is depicted in Fig. 1. At logistic regression analyses, adjusted for years it was observed that there was an increase in the frequency of *A. fumigatus* detection in June, (OR = 2.14, 95% CI [1.16; 3.98] *p* = 0.016) and a reduced frequency of isolation during September (OR = 0.48, 95% CI [0.27; 0.87] *p* = 0.015) and October as compared to January (OR = 0.39, 95% CI [0.21; 0.70] *p* = 0.002). At the same statistical analysis, seasonal trend for *A. niger* showed a reduced frequency of isolation from all clinical samples in the month of June as compared to January (OR = 0.34, 95% CI [0.14; 0.79] *p* = 0.012). Seasonal trend for *A. flavus* showed a higher frequency of detection in September (OR = 2.7, 95% CI [1.18; 6.18] *p* = 0.019), October (OR = 2.32, 95% CI [1.01; 5.35] *p* = 0.048) and November (OR = 2.42, 95% CI [1.01; 5.79] *p* = 0.047) as compared to January. No difference in seasonal frequency was observed for the other *Aspergillus* species.

For each *Aspergillus* species the number of isolates per year according to clinical settings and relative incidence rate for 10,000 patient-day are described in Table 2. An increase in incidence over time was reported only for LFI-*Aspergillus* spp., Mann Kendall trend test *p* = 0.0163. Confirmatory results were obtained at linear regression analyses run to investigate trends in incidence rates for each *Aspergillus* species individually and globally. According to these statistical analyses only LFI-*Aspergillus* species have showed a statistically significant [*r* = 0.36, (IC = 0.11;0.60, *p* < 0.05)] increase in incidence rates. Score test for trends of odds showed an overall increase in prevalence of LFI-*Aspergillus* species overtime, *p*-trend < 0.001. According to clinical settings, the same statistical analysis found an increase over time in prevalence of LFI-*Aspergillus* spp. in surgery *p*-trend = 0.0369, and in the outpatient clinic *p*-trend = 0.0004 although in both wards the number of LFI-*Aspergillus* spp. was lower than 10 per year. Also, prevalence of *A. niger* isolation throughout the study period increased in both ICU, *p*-trend = 0.0051 (Table 2), and BAL samples, *p*-trend = 0.0185 (Additional Table 1).

**Table 2:** *Aspergillus* spp. isolates per year according to clinical settings and relative incidence

		**N° of isolates**								
**Setting**	***Aspergillus* species**	**2015**	**2016**	**2017**	**2018**	**2019**	**2020**	**2021**	**2022**	overall
**Internal medicine**	***A. fumigatus***	22	24	18	25	29	18	11	22	169
	***A. niger***	8	14	6	6	10	2	4	8	58
	***A. flavus***	8	9	6	10	6	2	5	12	58
	***A. terreus***	3	7	5	3	5	1	4	8	6
	**others**	1	0	0	4	2	0	3	2	12
**Surgery**	***A. fumigatus***	4	12	3	4	3	2	2	5	35
	***A. niger***	3	5	3	3	4	2	3	2	25
	***A. flavus***	2	2	1	2	3	0	4	2	16
	***A. terreus***	0	1	0	0	2	0	1	0	4
	**others**	1	0	0	1	1	0	2	3	8
**ICU**	***A. fumigatus***	9	45	13	13	10	12	23	28	123
	***A. niger***	2	5	4	5	5	4	24	17	66
	***A. flavus***	7	6	6	9	8	4	10	16	66
	***A. terreus***	5	5	9	6	6	1	8	14	54
	**others**	1	1	4	0	1	1	3	2	13
**Outpatient clinic**	***A. fumigatus***	17	19	19	22	23	14	28	28	170
	***A. niger***	12	15	13	13	6	6	14	23	102
	***A. flavus***	2	3	8	15	5	5	5	16	59
	***A. terreus***	1	7	0	6	5	0	4	2	25
	**others**	0	1	1	0	4	4	10	7	27
**Incidence for 10,000 patient-day**	***A. fumigatus***	1.76	2.32	1.74	2.12	2.19	1.73	2.28	N/A^a^	
	***A. niger***	0.81	1.29	0.85	0.89	0.84	0.53	1.60	N/A	
	***A. flavus***	0.64	0.66	0,69	1.19	0.74	0.37	0.85	N/A	
	***A. terreus***	0.27	0.66	0.46	0.50	0.61	0.08	0.61	N/A	
	**others**	0.10	0.07	0.16	0.17	0.27	0.19	0.64	N/A	
	**Overall**	3.58	5.00	3.90	4.87	4.66	2.89	5.98	N/A	

^a^Data regarding 2022 patient-day have not been published yet

### Antifungal susceptibility test

**Fig. 2:**
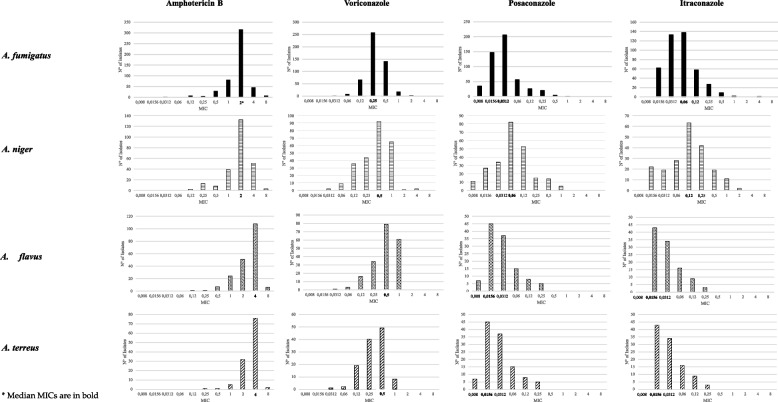
Antifungals MICs for the 4 most commonly isolated *Aspergillus* species. Each graph shows absolute number of isolates with corresponding MIC values. Median MIC are in bold. *A. fumigatus* is reported in full black color, *A. niger* horizontal bars, *A. flavus* black dots, and *A terreus* in diagonal bars

Distribution of MIC values for amphotericin B, voriconazole, posaconazole, and itraconazole are depicted in Fig. 2. MIC values and corresponding percentages of *A. fumigatus*, *A. niger*, *A flavus* and *A. terreus* isolates are reported in Table 3. MIC values and corresponding percentages of Less-Frequently-Isolated *Aspergillus* species (LFI-*Aspergillus* spp.) are shown in Table 4.

**Table 3:** Percentage of antifungals MICs for *Aspergillus* species with ECVs

Species	Antifungal agent	% of isolates with MIC (µg/mL) of											
		≤ 0.008	0.0156	0.0312	0.06	0.12	0.25	0.5	1	2	4	≥ 8	ECV (µg/mL)
***A. fumigatus***	AMB	0.0	0.0	0.4	0.0	1.6	0.8	5.8	16.5	63.7	**9.3**	**1.6**	2
	VRC	0.0	0.0	0.4	1.6	13.5	51.9	28.4	3.6	**0.6**	0.0	0.0	1
	PSC	7.1	29.7	41.6	11.3	5.3	4.0	**0.8**	**0.2**	0.0	0.0	0.0	0.5
	ITR	0.0	14.4	30.9	32.1	13.5	6.3	2.1	0.5	0.0	**0.2**	0.0	1
***A. niger***	AMB	0.0	0.0	0.0	0.0	0.8	5.2	3.2	15.7	53.2	**20.6**	**1.2**	2
	VRC	0.0	0.0	0.8	3.6	11.2	20.3	36.7	25.9	0.4	0.0	**0.8**	2
	PSC	4.6	11.2	14.1	34.0	22.0	6.2	5.8	2.1	0.0	0.0	0.0	2
	ITR	0.0	10.7	9.2	13.6	30.6	20.4	9.2	5.3	1.0	0.0	0.0	4
***A. flavus***	AMB	0.0	0.0	0.0	0.0	0.5	0.5	3.5	12.1	25.8	54.5	**3.0**	4
	VRC	0.0	0.0	0.5	1.5	7.5	17.6	39.7	30.7	2.0	**0.5**	0.0	2
	PSC	3.1	5.6	10.8	40.5	22.1	12.8	3.1	**2.1**	0.0	0.0	0.0	0.5
	ITR	0.0	9.9	15.2	35.1	21.1	14.6	3.5	0.0	0.0	**0.6**	0.0	1
***A. terreus***	AMB	0.0	0.0	0.0	0.0	0.0	0.9	0.9	4.3	27.4	65.0	**1.7**	4
	VRC	0.0	0.0	0.8	1.7	16.0	33.61	41.2	6.7	0.0	0.0	0.0	2
	PSC	6.0	38.5	31.6	12.8	6.8	4.3	0.0	0.0	0.0	0.0	0.0	1
	ITR	0.0	41.0	32.4	15.2	8.6	2.9	0.0	0.0	0.0	0.0	0.0	2

MIC values above the corresponding ECV are in bold

*AMB* amphotericin B, *VRC* voriconazole, *PSC* posaconazole, *ITR* itraconazole

**Table 4:** Percentage of antifungals MICs for *Aspergillus* species without ECV values

Species	Antifungal agent	% of isolates with MIC (µg/mL) of											
		≤ 0.008	0.0156	0.0312	0.06	0.12	0.25	0.5	1	2	4	≥ 8	ECV (µg/mL)
*A. nidulans*	AMB							17.4	13.0	34.8	30.4	4.3	N/A
	VRC			4.3	30.4	17.4	17.4	21.7	8.7				N/A
	PSC	4.3	26.1	21.7	26.1	4.3	17.4						N/A
	ITR		26.1	30.4	17.4		13.0	4.3					N/A
*A. candidus*	AMB								20	40	40		N/A
	VRC					10	20	40	10	20			N/A
	PSC	10	20	30	20	10	10						N/A
	ITR		20	40		0	10	10					N/A
*A. usutus*	AMB										100		N/A
	VRC							100					N/A
	PSC			100									N/A
	ITR			100									N/A
*A. glaucus*	AMB											2	N/A
	VRC								50			50	N/A
	PSC					50						50	N/A
	ITR						50					50	N/A
*A. oryziae*	AMB											100	N/A
	VRC									100			N/A
	PSC								100				N/A
	ITR								100				N/A
*A. clavatus*	AMB							100					N/A
	VRC								100				N/A
	PSC		100										N/A
	ITR			100									N/A
*A. versicolor*	AMB							14.3		14.3	**14.3**	**14.3**	2
	VRC				57.1	14.3	28.6						N/A
	PSC		14.3	42.9		28.6	14.3						N/A
	ITR		14.3	28.6	28.6								N/A
*A. lentulus*	AMB							16.7	50.0	16.7			N/A
	VRC					33.3	50.0	16.7					N/A
	PSC	16.7	33.3	33.3	16.7								N/A
	ITR		50.0		16.7								N/A
*A. scleroticum*	AMB						25	50					N/A
	VRC			12.5	25		50	12.5					N/A
	PSC		12.5	37.5		12.5	12.5	12.5					N/A
	ITR												N/A
*A. ochraceus*	AMB										100		N/A
	VRC							100					N/A
	PSC					100							N/A
	ITR						100						N/A

MIC values above the corresponding ECV are in bold

*AMB* amphotericin B, *VRC* Voriconazole, *PSC* Posaconazole, *ITR* itraconazole

*Amphotericin B*. Percentages of isolates with MIC values above the ECVs were: 21.8% for *A. niger*; 10.9% for *A. fumigatus*; 3% for *A. flavus*; 1.7% for *A. terreus*.

*Voriconazole*. Percentages of isolates with MIC values above the ECVs were: 0.6% for *A. fumigatus*; 0.8% for *A. niger*; 0.5% for *A. flavus*; 0% for *A. terreus*.

*Posaconazole*. Percentages of isolates with MIC values above the ECVs were: 1% for *A. fumigatus*; 2.1% for *A. flavus*; 0% for *A. niger* and *A. terreus*.

*Itraconazole*. Percentages of isolates with MIC values above the ECVs were: 0.2% for *A. fumigatus*; 0.6% for *A. flavus*; 0% for *A. niger* and *A. terreus*.

ECV values are not available for LFI-*Aspergillus* spp. with the exception of amphotericin B for *A. versicolor*; in this case, 1 (14.3%) isolate reported a MIC = 4 mg/L and 1 (14.3%) a MIC ≥ 8 mg/L.

No statistically significant increase in azole and amphotericin resistance was observed over time at both score test for trend of odds and logistic regression analyses (*p*-value > 0.05).

## Discussion

This surveillance study evaluated the prevalence, species distribution, seasonality, time trends, and antifungal susceptibility profiles of all *Aspergillus* spp. isolates recovered in our clinical database since its implementation in 2015 up to 2022. *Aspergillus fumigatus* was confirmed to be the first *Aspergillus* species isolated from all registered clinical samples and in every clinical setting. However, surprisingly, despite being the most prevalent species registered, no increase in both its prevalence and incidence was observed overtime. On the contrary, prevalence of isolated *A. niger* increased in ICU and particularly from BAL samples. Similar trend was reported in the prevalence of LFI-*Aspergillus* species showing an increase in both prevalence and incidence over the 8-year study period across all clinical settings. According to clinical settings, risk of detection of *A. terreus* was higher in ICU rather than internal medicine. Instead, outpatient clinic was associated with a higher risk of detecting *A. niger*, as well as risk of LFI-*Aspergillus* spp. detection was higher in surgery. To this point, it is mandatory to highlight that the highest number of *Aspergillus* isolates was surprisingly recovered from the outpatient clinic (34% of all isolates). This could be explained by the fact that the pediatric cystic fibrosis service was a consistent part in the outpatient clinic. Indeed, since this special outpatient unit manages patients at higher risk of contracting *Aspergillus* colonization and/or infection, it registered alone 273 cases (71.3%) – data not shown – out of the 383 *Aspergillus* isolates recovered from the entire clinical setting.

Data on seasonal frequency trends found a significant increase in the frequency of *A. fumigatus* detection in June and a reduction in September and October as compared to January across the 8-year study period recorded in the database. Parallel to this, frequency of *A. niger* detection was lower in June. The same seasonality analysis found a higher frequency of detection of *A. flavus* from September to November.

Respiratory samples are the predominant clinical samples, in fact, the vast majority (86.1%) of *Aspergillus* species was recovered from either BAL or sputum.

When focusing on the MIC distribution for each tested antifungal, this study shows a striking low prevalence of non-WT isolates for azole compounds. Unexpectedly, no isolate had a non-WT phenotype for all the azoles tested. The percentages of *A. fumigatus* with a non-WT phenotype for voriconazole, posaconazole and itraconazole were 0.6%, 1% and 0.2% respectively. Similar low prevalence in non-WT phenotypes for all the azole compounds analyzed was observed for *A. niger* (0.8% of isolates with non-WT MIC for voriconazole), *A. flavus* (0.5% of non-WT isolates for voriconazole, 2.1% for posaconazole and 0.6% for itraconazole), and *A. terreus* with no reported MIC above the ECVs. Despite low prevalence of non-WT phenotypes for azole compounds screening for genetic mechanisms as CYP51A for azole resistance in the non-WT population will be a future task. Opposed to these low percentages, prevalence of non-WT MIC for amphotericin B was higher in all the analyzed species. It is intriguing to notice that *A. niger* reported the highest prevalence of non-WT phenotypes for amphotericin B (21.8%), followed by *A. fumigatus* (10.9%), *A. flavus* 3% and *A. terreus* 1.7%.

Comparing the results reported in this study with other studies published it appears reasonable to state that prevalence and species distribution of commonly isolated *Aspergillus* species have similar rates and trends to those reported in literature [42]. Of notice, *A. flavus* is reported to be the second clinical isolate in terms of prevalence from several studies [42, 43], however in this epidemiological study it was the third most common isolate after *A. fumigatus* and *A. niger.* Respiratory specimens could be still considered the most frequent clinical samples from which the majority of *Aspergillus* spp. are recovered [8, 13]. On the other hand, seasonal trends might be difficult to compare as they have only been largely studied in environmental research, but not within a single hospital environment [44–46], as hospital outbreaks of IA have been related to works of construction, renovation and/or demolition happening within the hospital or in the nearby surrounding areas [47]. According to these environmental reports, lower incidence was reported for all species in January, March and February but still they are not hospital related [44]. Anyway, what was carried out in this study was a different seasonality profile in *Aspergillus* spp. isolation within the hospital environment than those reported in the above cited environmental studies.

In addition, it is intriguing to notice that data regarding antifungal susceptibility tests and MIC distribution for azole compounds in this study seemed not to reflect the current increase in azole resistance, as rates of non-WT isolates appeared to be lower than the ones reported in literature up to date [28, 48]. In fact, even if establishing relative prevalence or non-WT isolate rates among *Aspergillus* species worldwide could be a difficult task to achieve [28], several reports show that azole resistance rates and/or non-WT phenotypes in *A. fumigatus* range from 7.5% to 10.9% in environmental isolates and from 3.4% to 17.8% in clinical ones [42, 48–51]. These percentages resulted to be higher than those reported in this study.

A specific mention should be stated regarding prevalence of amphotericin B non-WT isolates. Despite resistance mechanisms for this drug in *A. fumigatus*, *A. niger* and *A. flavus* are still a matter of debate and have still to be defined, multiple reports and meta-analysis have investigated this topic through time [29, 52–55]. In this study, *Aspergillus* species isolates registered the highest prevalence of non-WT amphotericin B MIC among all the tested antifungal compounds. *A. fumigatus* and *A. flavus* had a 10.9% and 3% of non-WT phenotypes respectively. Unexpectedly, *A niger* was the species with the highest prevalence of non-WT phenotypes for amphotericin B with 21.8% isolates with MIC values above the ECVs. These findings appear to be in contrast with current literature, where prevalence of non-WT isolates for amphotericin B was higher for *A. flavus* (14.9%—66.6%) and lower for *A. niger* (5.2%) and *A. fumigatus* (2%) [42, 53].

These results should be anyhow interpreted with caution. This study is a retrospective analysis of all isolates reported in our database. One major drawback of the present study is the limited diagnostic accuracy from 2015 to 2019 where only macroscopic and microscopic examinations were the cornerstones of *Aspergillus* spp. identification. Consequently, it must be pointed out that the increase in the LFI-*Aspergillus*-species could have been substantially influenced by the implementations applied in the diagnostic process from 2020, where macroscopic and microscopic examinations were followed by MALDI TOF analyses. Due to the retrospective nature of the study, the data on *Aspergillus* spp. isolates were collected from a microbiology laboratory database, molds could not be stored in frozen livestock, therefore could not be retested with MALDI TOF to confirm prior identification. Unfortunately, no clinical information regarding patients’ preexisting conditions and/or treatment plus clinical evolution were available. In addition, no effective diagnosis of invasive aspergillosis regarding each isolate could be assumed when looking at the data, as this process involves clinical picture, patient’s history, imaging and serology. Same assumptions can be applied to the outpatient samples. Based only on the information stored in the microbiological database distinguishing the role of the isolated mold between contaminant or causative agent was beyond our possibilities. However, even in this particular clinical scenario, all processed samples were taken from anatomical sites that could be at high risk of developing invasive aspergillosis. This point limited also the investigation on patients’ travel history, as this aspect would have been worthy of evaluation when trying to explain the peculiar seasonal trend of *Aspergillus flavus* that appeared to be more pronounced in September, October and November, as these months usually follow vacations period. Since this species is predominant in the tropical areas, history of recent travel to such places might be an explanatory factor of this seasonal trend. Last but not least, no further molecular investigation regarding possible mutations on outlier isolates with MIC above the ECV could be afforded. Such an investigation would have prompted a useful correlation between non-WT MICs and eventual treatment outcomes. Another issue regarding antimicrobial susceptibility test results must be addressed, as from 2015 to 2019 identification of *Aspergillus* spp. relied only on macroscopic and microscopic examination and therefore species identification might have suffered a lack in diagnostic performance. This could have led in some cases to misidentification and consequently might have affected antifungal susceptibility test interpretation. However, by looking at the data before and after the implementation of MALDI TOF, same susceptibility phenotypes have all been confirmed reporting higher rates for AMB non-WT phenotypes and extremely low prevalence of non-WT isolates for azole compounds.

Despite these limitations, the large time span, the elevated number of *Aspergillus* isolates, the longitudinal collection of data, all data regarding antifungal susceptibility, and the relative low number of missing values are all points of strength of this study.

Future studies should aim at investigating correlation between hospital epidemiology, antifungal susceptibility testing of IA and correlation with clinical outcomes.

## Conclusions

To the best of our knowledge, this is the first surveillance study to ever compare and include at once data regarding prevalence, time trends, seasonality, species distribution and antifungal susceptibility profiles of all *Aspergillus* spp. isolates ever recorded in a clinical database since its implementation in clinical practice.

### Supplementary Information

**Supplementary Material 1. **

## Data Availability

The datasets used and/or analyzed during the current study are available from the corresponding author on reasonable request. Total patient-days data per year were obtained from hospital publicly available repository https://www.ao-pisa.toscana.it/index.php?option=com_content&view=article&id=5650:relazione-sanitaria-2021&catid=264&Itemid=112
