# Supplementary material for: Hospital distribution, seasonality, time trends and antifungal susceptibility profiles of all Aspergillus species isolated from clinical samples from 2015 to 2022 in a tertiary care hospital
Source: BMC Microbiol. 2024 Apr 3;24:111. doi: 10.1186/s12866-024-03267-8 (PMC10988875; doi:10.1186/s12866-024-03267-8)
Supplement: Supplementary file 1 — Supplementary Material 1. [file 12866_2024_3267_MOESM1_ESM.docx]

**Supplementary table 1**. *Aspergillus* spp. isolates per year according to clinical samples.

|  | *Aspergillus fumigatus* | | | *Aspergillus niger* | | | *Aspergillus terreus* | | | *Aspergillus flavus* | | | LFI-*Aspegillus* spp. | | |
| --- | --- | --- | --- | --- | --- | --- | --- | --- | --- | --- | --- | --- | --- | --- | --- |
| **year** | **Sputum** | **BAL** | **others*** | **Sputum** | **BAL** | **others*** | **Sputum** | **BAL** | **others*** | **Sputum** | **BAL** | **others*** | **Sputum** | **BAL** | **others*** |
| 2015 | 34 | 14 | 4 | 14 | 6 | 5 | 2 | 7 | 0 | 8 | 8 | 1 | 1 | 2 | 0 |
| 2016 | 30 | 23 | 17 | 21 | 9 | 7 | 7 | 11 | 2 | 8 | 7 | 5 | 1 | 1 | 0 |
| 2017 | 24 | 23 | 6 | 14 | 7 | 5 | 0 | 13 | 1 | 6 | 7 | 8 | 1 | 4 | 0 |
| 2018 | 38 | 22 | 4 | 16 | 6 | 5 | 7 | 7 | 1 | 18 | 13 | 5 | 0 | 3 | 2 |
| 2019 | 35 | 21 | 9 | 11 | 7 | 7 | 8 | 7 | 3 | 8 | 11 | 3 | 6 | 1 | 1 |
| 2020 | 23 | 18 | 5 | 7 | 4 | 3 | 0 | 2 | 0 | 4 | 6 | 1 | 4 | 0 | 1 |
| 2021 | 32 | 29 | 3 | 17 | 25 | 3 | 7 | 10 | 0 | 9 | 10 | 5 | 10 | 5 | 3 |
| 2022 | 38 | 36 | 9 | 20 | 23 | 7 | 4 | 19 | 1 | 21 | 18 | 7 | 7 | 3 | 4 |
| total | 254 | 186 | 57 | 120 | 87 | 44 | 35 | 76 | 8 | 82 | 80 | 37 | 30 | 19 | 11 |

*Others : Wound/Biopsy; Ear Swab; Nasal Swab; Eye Swab; Sinus Discharge; Pleural Fluid, Cerebro-spinal Fluid. BAL=Bronchoalveolar Lavage Fluid. BAL= Bronchoalveolar Lavage Fluid.
